# The design of schistosomiasis monitoring and evaluation programmes: The importance of collecting adult data to inform treatment strategies for *Schistosoma mansoni*

**DOI:** 10.1371/journal.pntd.0006717

**Published:** 2018-10-08

**Authors:** Jaspreet Toor, Hugo C. Turner, James E. Truscott, Marleen Werkman, Anna E. Phillips, Ramzi Alsallaq, Graham F. Medley, Charles H. King, Roy M. Anderson

**Affiliations:** 1 London Centre for Neglected Tropical Disease Research and Department of Infectious Disease Epidemiology, School of Public Health, Faculty of Medicine, St Mary’s Campus, Imperial College London, London, United Kingdom; 2 Oxford University Clinical Research Unit, Wellcome Trust Major Overseas Programme, Ho Chi Minh City, Vietnam; 3 Centre for Tropical Medicine and Global Health, Nuffield Department of Medicine, University of Oxford, Oxford, United Kingdom; 4 The DeWorm3 Project, The Natural History Museum of London, London, United Kingdom; 5 Center for Global Health and Diseases and Department of Mathematics, Case Western Reserve University, Cleveland, Ohio, United States of America; 6 Centre for Mathematical Modelling of Infectious Disease, London School of Hygiene and Tropical Medicine, London, United Kingdom; Johns Hopkins University, UNITED STATES

## Abstract

Monitoring and evaluation (M&E) programmes are used to collect data which are required to assess the impact of current interventions on their progress towards achieving the World Health Organization (WHO) goals of morbidity control and elimination as a public health problem for schistosomiasis. Prevalence and intensity of infection data are typically collected from school-aged children (SAC) as they are relatively easy to sample and are thought to be most likely to be infected by schistosome parasites. However, adults are also likely to be infected. We use three different age-intensity profiles of infection for *Schistosoma mansoni* with low, moderate and high burdens of infection in adults to investigate how the age distribution of infection impacts the mathematical model generated recommendations of the preventive chemotherapy coverage levels required to achieve the WHO goals. We find that for moderate prevalence regions, regardless of the burden of infection in adults, treating SAC only may achieve the WHO goals. However, for high prevalence regions with a high burden of infection in adults, adult treatment is required to meet the WHO goals. Hence, we show that the optimal treatment strategy for a defined region requires consideration of the burden of infection in adults as it cannot be based solely on the prevalence of infection in SAC. Although past epidemiological data have informed mathematical models for the transmission and control of schistosome infections, more accurate and detailed data are required from M&E programmes to accurately determine the optimal treatment strategy for a defined region. We highlight the importance of collecting prevalence and intensity of infection data from a broader age-range, specifically the inclusion of adult data at baseline (prior to treatment) and throughout the treatment programme if possible, rather than SAC only, to accurately determine the treatment strategy for a defined region. Furthermore, we discuss additional epidemiological data, such as individual longitudinal adherence to treatment, that should ideally be collected in M&E programmes.

## Author summary

Schistosomiasis remains an endemic parasitic disease affecting millions of people around the world. The World Health Organization (WHO) has set goals of morbidity control and elimination as a public health problem for schistosomiasis defined by reaching ≤5% and ≤1% prevalence of heavy-intensity infections in school-aged children, respectively. Monitoring and evaluation (M&E) programmes are used to collect data which can inform treatment strategies required in a defined area and can also aid in assessing the progress of implemented treatment strategies. Due to programmatic and financial constraints, M&E data are typically collected from school-aged children as they are thought to be most likely to be infected. We highlight that adults should be included within M&E programmes by showing how the burden of infection in adults impacts our mathematical model recommendations of the treatment coverage levels required to reach the WHO goals for schistosomiasis. Our results highlight the importance of collecting data from a broader age-range, specifically the inclusion of adult data at baseline (prior to treatment) and throughout the treatment programme if possible. Improving M&E programmes to incorporate collection of such data will allow for more accurate determination of the optimal treatment strategy for a defined region.

## Introduction

Schistosomiasis is a neglected tropical disease (NTD) caused by parasitic worms which may infect the intestines (*Schistosoma mansoni* or *S*. *japonicum)* or urinary tract *(S*. *haematobium)*. The disease is endemic in 54 countries affecting approximately 240 million people worldwide [1]. The World Health Organization (WHO) has set goals of morbidity control and elimination as a public health problem, i.e. reaching ≤5% and ≤1% prevalence of heavy-intensity infections in school-aged children (SAC; 5–14 years of age), respectively [2]. The overall aim is to achieve these goals using mass drug administration (MDA) of praziquantel at the treatment frequency and coverage level recommended by the WHO [1, 2]. The current WHO recommendations are treatment of SAC once every 3 years in low prevalence settings (<10% baseline prevalence among SAC), and treatment of SAC as well as adults considered to be at risk of infection once every 2 years in moderate prevalence settings (10–50% baseline prevalence among SAC) and once a year in high prevalence settings (≥50% baseline prevalence among SAC) [1, 2]. Recent analyses based on mathematical models of schistosome transmission have recommended adaptations to these guidelines, such as an increase and expansion in treatment coverage levels to include adults or an increase in treatment frequency [3].

As SAC are most likely to be infected by *Schistosoma* parasites, possibly due to age-related water contact behaviour and/or the absence of acquired immunity generated by repeated exposure to infection, treatment has been specifically focused at this age group to prevent morbidity [4–6]. Given the high prevalence and intensity of infection typically in SAC, treating this age group may be more efficient and may have a greater impact on reducing transmission to the rest of the community. School-based treatment has been widely used as SAC are relatively easy to locate, sample and therefore reach good coverage levels. This makes SAC highly important in terms of monitoring the impact of MDA treatment. By 2020, WHO aims to increase coverage such that 75% of SAC at risk will be regularly treated in endemic countries [7]. However, adults (ages 15+ as defined by WHO) are also likely to be infected [8, 9]. Hence, it is also important to include them in treatment and monitoring efforts since if MDA is only targeted at SAC, a large proportion of the local *Schistosoma* infection burden will remain untreated by chemotherapy creating a reservoir of infection within the community [8, 9]. In 2002, it was recommended that adults be treated in high-risk areas and that women of childbearing age not be excluded from MDA coverage [10]. However adult treatment has not been regularly implemented in most MDA programmes [1, 3, 8]. MDA coverage data collated by WHO reflect this situation [11]. Pre-school aged children (pre-SAC) may also be infected, though at present they are not eligible for treatment with praziquantel [12]. However, recent work shows that praziquantel may be used to treat pre-SAC provided the dosage is correct [13] so these guidelines may change in the coming years.

The WHO recognizes monitoring and evaluation (M&E) as an essential component of any treatment programme, and recommends the regular use of process and performance indicators for monitoring (i.e. assessing organizational elements and coverage levels), and performance and impact indicators for evaluation (i.e. assessing coverage levels, prevalence and intensity of infection) [1, 2]. It is vital that well-designed M&E activities are carried out to collect data that can assist in determining the progress of MDA programmes. This can give insight into the degree to which the prevalence and/or intensity of infection in a region has been reduced following treatment. Due to financial and programmatic constraints, data is typically collected from SAC only as they are most likely to be infected but also treated by national control programmes [14, 15]. Additionally, the preventive chemotherapy (PCT) strategy for a region is currently dependent upon the prevalence of infection in SAC at baseline (prior to treatment) with the WHO goals being dependent upon the prevalence of heavy-intensity infections in SAC only [1]. However, the prevalence of infection in SAC has been an inconsistent indicator of the burden of infection in adults [15–19]. The burden of infection in adults will vary geographically as it is likely to be impacted by local behavioural and cultural factors, as well as access to water, sanitation and hygiene (WASH) facilities [17].

The age profile of infection and more specifically, the burden of infection in adults relative to SAC, has been observed to vary for schistosome infections in different countries and communities [8, 17, 20–23]. In this study, we explore how different age profiles of infection with low to high burdens of infection in adults can impact our mathematical model generated recommendations of the treatment coverage levels required to achieve the WHO goals for *S*. *mansoni*. We show the importance of collecting broader age-specific data, particularly including adults as well as SAC, within M&E programmes. We also offer guidance on how M&E programmes should be designed by highlighting other data required to determine the progress of treatment strategies.

## Methods

### Model

**Fig 1:**
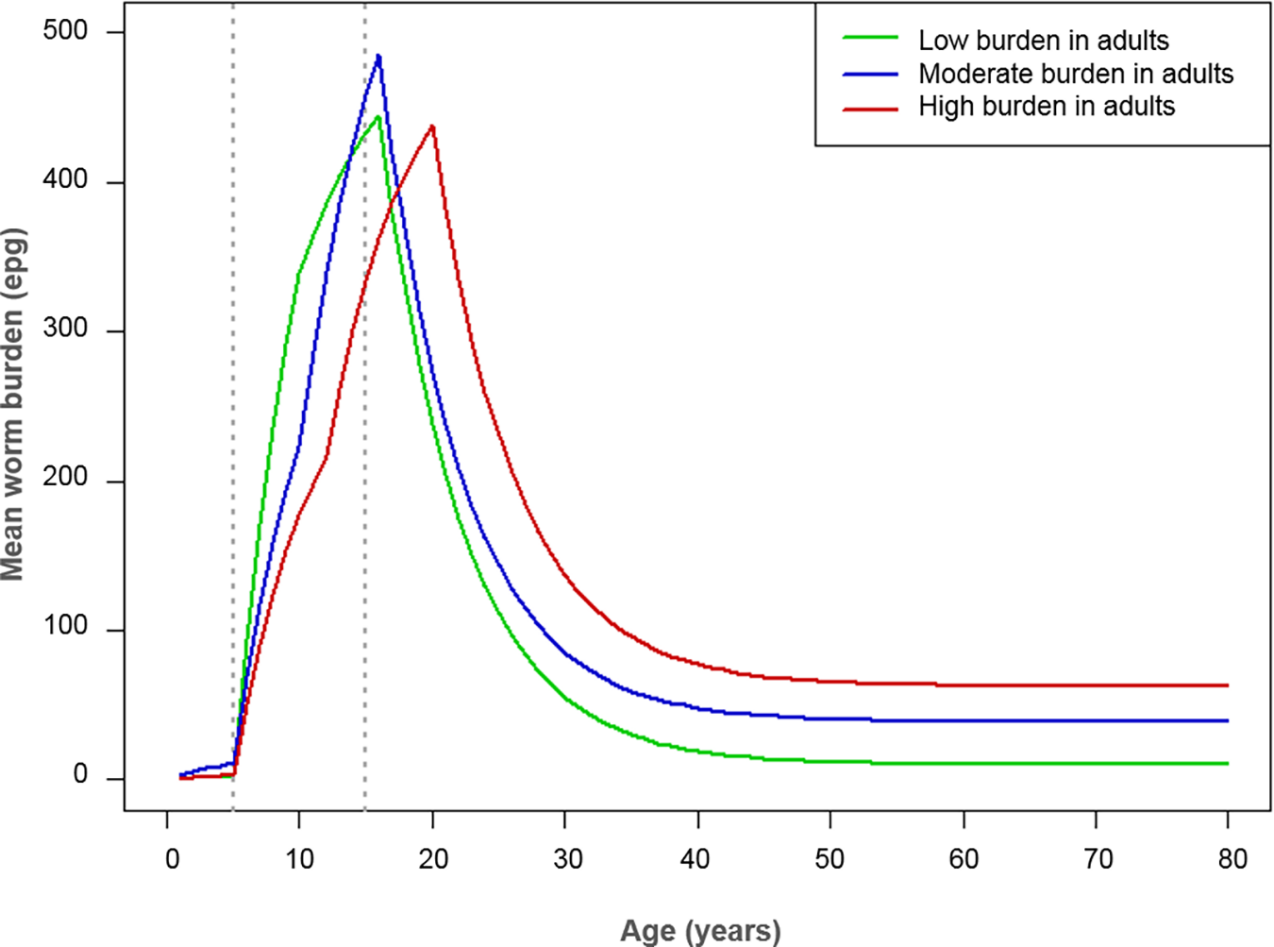
Age-intensity profiles of infection. Mean worm burden (eggs per gram of faeces, epg) across ages with low, moderate and high burdens of infection in adults relative to school-aged children (school-aged children are between 5–14 years of age and adults are 15+ years of age) [17].

We used an age-structured deterministic model of parasite transmission and control by praziquantel treatment developed by Imperial College London [8, 24] to follow through the currently recommended WHO guidelines for *S*. *mansoni* [2] (it is important to note that previous model analyses have suggested important adaptations to these guidelines [3]). Model parameters were based on previous studies (parameter values are shown in Table 1). To determine whether the burden of infection in adults relative to SAC would have an impact on the recommended treatment coverage levels, we used three different age-intensity profiles of infection which had a low, moderate or high burden of infection in the adult population. The age-intensity profiles were simulated in line with past epidemiological studies [21, 25, 26] and produced by varying the age-specific contact rates, i.e. the transmission intensity by age group, as shown in Fig 1 and Table 1 [17]. The prevalence in adults varied depending on the age-intensity profile of infection, i.e. as the burden of infection in adults relative to SAC increased, the peak of the age profile shifted to older age groups and the prevalence of infection in adults rose (baseline prevalence for adults shown in S1–S3 Tables). We then determined the coverage levels required to reach the WHO goals of morbidity control and elimination as a public health problem within a 5, 10 and 15-year programme.

**Table 1:** Parameter values used within the age-structured deterministic model for *Schistosoma mansoni* (based on previous studies).

Parameter	Value	Source
Fecundity	0.34 eggs/female/sample	[27–29]
Egg distribution within the individual	0.87	[28, 29]
Aggregation parameter	0.04 (baseline SAC prevalence settings close to 10%); 0.24 (baseline SAC prevalence settings > 10%)	[8, 30]
Density dependence fecundity	0.0007/female worm	[8, 17]
Worm lifespan	5.7 years	[21, 27]
Drug efficacy	86.3%	[31]
Low adult burden setting: Age specific contact rates for 0–5, 5–10, 10–16, 16+ years of age	0.01, 1.2, 1, 0.02	[17]
Moderate adult burden setting: Age specific contact rates for 0–5, 5–10, 10–16, 16+ years of age	0.032, 0.61, 1, 0.06	[17]
High adult burden setting: Age specific contact rates for 0–5, 5–12, 12–20, 20+ years of age	0.01, 0.61, 1, 0.12	[17]
Prevalence of infection	Percentage of population having egg count threshold (or eggs per gram, epg) > 0	-
Prevalence of heavy-intensity infections	Percentage of population having egg count threshold ≥ 16 (epg ≥ 400 divided by 24 to convert to egg count)	[32]
Human demography	Based on Uganda’s demographic profile	[33, 34]

### Simulated treatment strategy

Beginning with an untreated population (run for 1000 years prior to initiating treatment in order to reach the endemic phase at a stable equilibrium), we implemented the frequency of PCT depending on the baseline prevalence in SAC (estimated by using Kato-Katz as the diagnostic test as currently recommended by WHO [2]). We carried out PCT as recommended by WHO at a 75% coverage level of SAC only whilst assuming 100% treatment adherence (with the drug efficacy of praziquantel set at 86.3% [31]). For moderate prevalence settings (10–50% SAC baseline prevalence), we carried out treatment once every 2 years and for high prevalence settings (≥50% SAC baseline prevalence), we carried out treatment once a year. To simulate a range of baseline prevalence levels falling within these settings, the intrinsic intensity of transmission, i.e. basic reproductive number (R_0_) [8], was varied in our model (such that, the prevalence at baseline increased with R_0_; R_0_ values shown in S1–S3 Tables). The aggregation parameter (k) was varied to define different prevalence levels at a stable equilibrium prior to treatment, i.e. lower baseline prevalence settings were reached using a lower k value reflecting higher degrees of parasite aggregation in the human host (k values shown in Table 1).

### Model output

Following 5, 10 and 15 years of PCT, we determined the prevalence of heavy-intensity infections in SAC to investigate whether the WHO goals had been achieved (where the morbidity and elimination as a public health problem goals are reached once this prevalence is ≤5% and ≤1% in SAC, respectively). In cases where the WHO goals were not achievable within 5 to 15 years treatment of SAC only, we increased the level of SAC coverage and included adult treatment to determine the levels of coverage that would be required to make the goals achievable. Throughout the simulations of the deterministic model, we projected both the prevalence of infection (eggs per gram, epg > 0) and prevalence of heavy-intensity infections (epg ≥ 400; [1, 32]) in SAC and adults.

## Results

**Fig 2:**
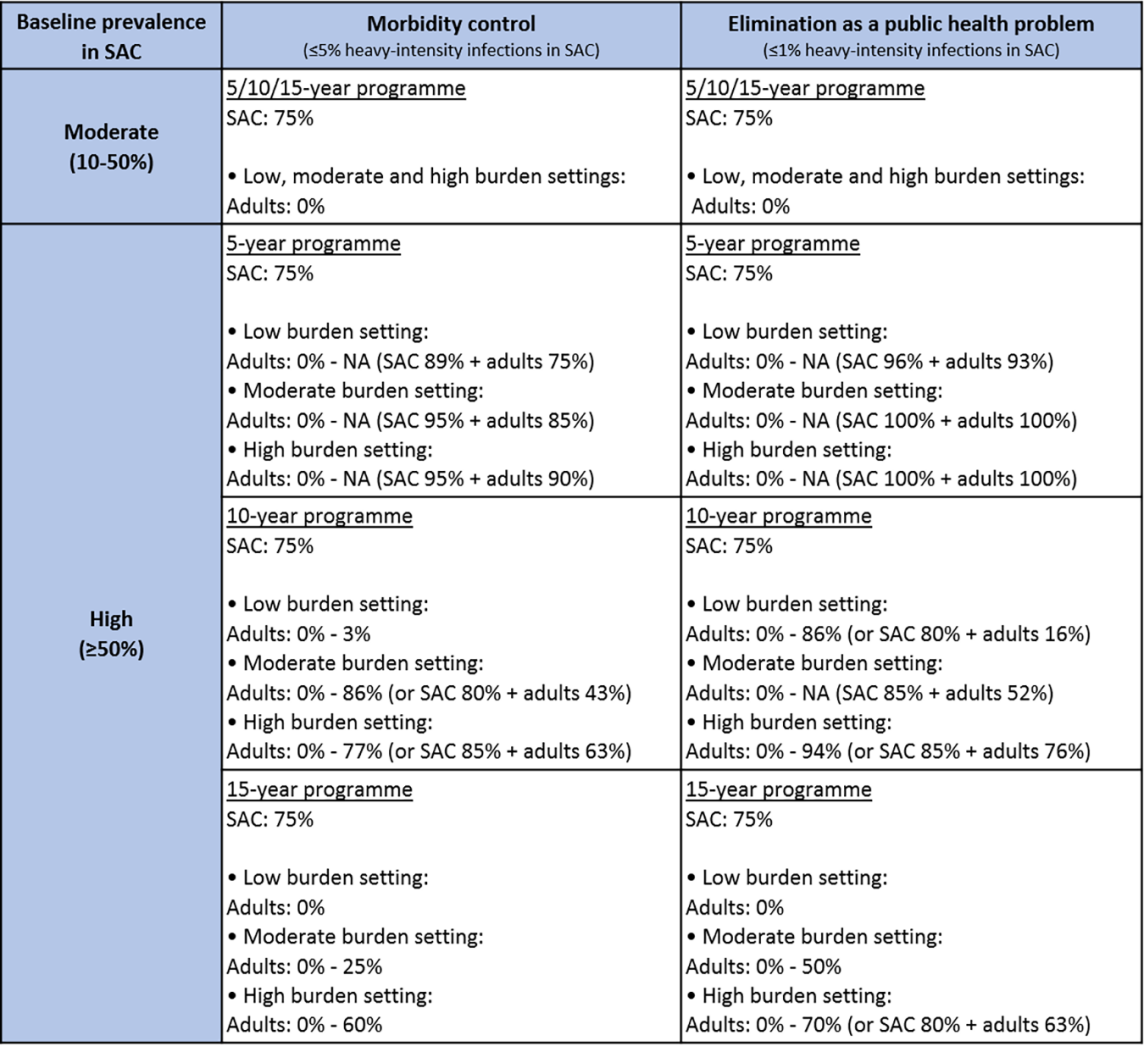
Coverage levels required to meet the World Health Organization (WHO) goals. Levels of school-aged children (SAC; 5–14 years of age) and adult (≥ 15 years of age) coverage required to meet the WHO goals when following currently recommended WHO treatment frequencies, i.e. for moderate baseline prevalence in SAC, treating once every 2 years; for high baseline prevalence in SAC, treating once a year. Required coverage levels are shown for a 5, 10 and 15-year treatment programme within low, moderate and high adult burden of infection settings. NA: not achievable unless SAC coverage is increased above 75%. More detailed S1–S3 Tables.

**Fig 3:**
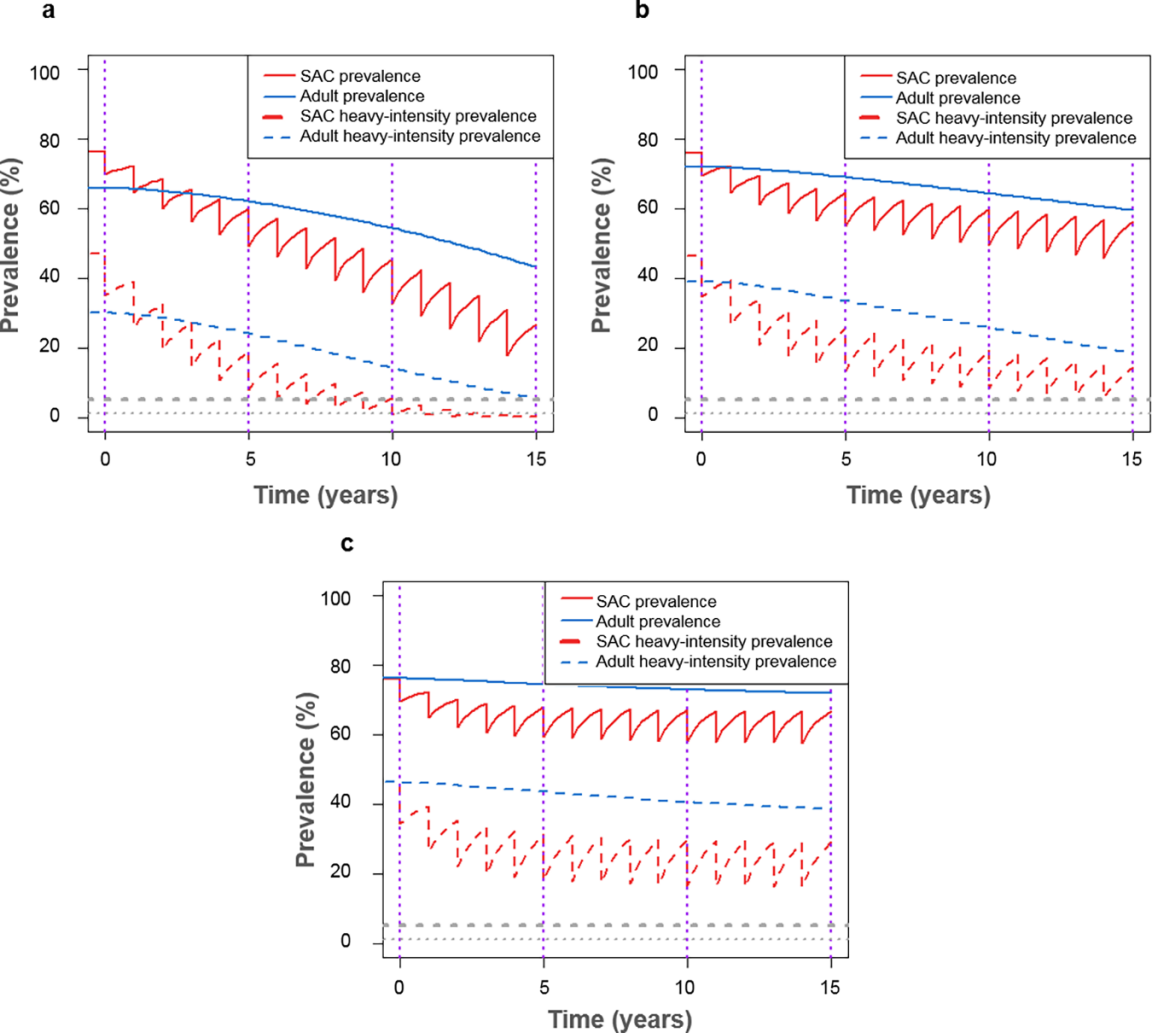
Model projections of annual treatment of 75% school-aged children (SAC; 5–14 years of age) and 0% adults (15+ years of age). Shown for high prevalence settings (≥50% SAC baseline prevalence) for (a) low (R_0_ = 3.0), (b) moderate (R_0_ = 3.5) and (c) high (R_0_ = 4.0) adult burdens of infection.

**Fig 4:**
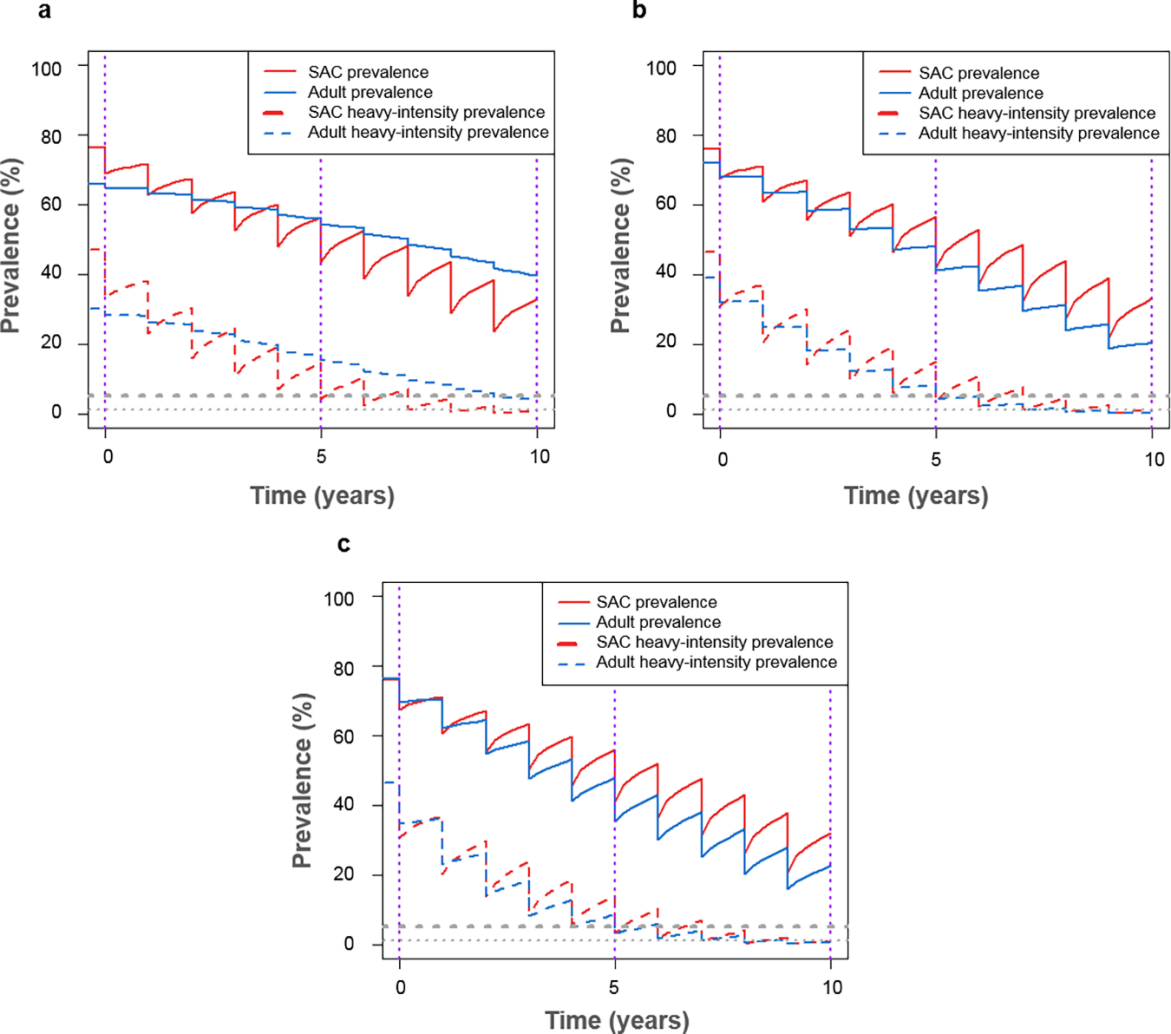
Model projections of annual treatment for high prevalence settings (≥50% SAC baseline prevalence) using coverage levels required to achieve elimination as a public health problem within 10 years. With (a) low adult burden of infection (R_0_ = 3.0) treating 80% school-aged children (SAC; 5–14 years of age) and 16% adults (15+ years of age), (b) moderate adult burden of infection (R_0_ = 3.5) treating 85% SAC and 52% adults, and (c) high adult burden of infection (R_0_ = 4.0) treating 85% SAC and 76% adults.

In moderate prevalence settings, i.e. baseline prevalence 10–50% in SAC, we found that the WHO goals were likely to be achieved within 5 years following biennial treatment of 75% SAC only. This held regardless of whether the burden of infection in adults was low, moderate or high, as the WHO goals remained achievable with no adult treatment (Fig 2). However, for high prevalence settings (baseline prevalence ≥50% in SAC), we found that the WHO goals were not likely to be achieved within 5 to 15 years following annual treatment with coverage at 75% of SAC only (Fig 3; aligning with previous work [3]). In these higher prevalence regions, treatment of adults as well as SAC was required within the treatment programme, with coverage levels varying with the burden of infection in adults (Fig 4; though for baseline prevalence levels close to 50% in SAC, the goals were achievable with 75% SAC only treatment).

Given a high burden of infection in adults, the baseline prevalence of infection and heavy-intensity infections in adults was higher than SAC (Fig 3). Following treatment of SAC only, the prevalence of infection and heavy-intensity infections in SAC declined but remained high in adults, regardless of the burden of infection in adults (Fig 3). When not included within the treatment programme, infected adults remained as a reservoir of infection, making it vital that they are included within treatment. Expanding to include adult treatment led to decreased prevalence of infection and heavy-intensity infections in adults and simultaneously had a beneficial impact on further reducing the prevalence in SAC relative to treating SAC only (Figs 3 and 4).

**Fig 5:**
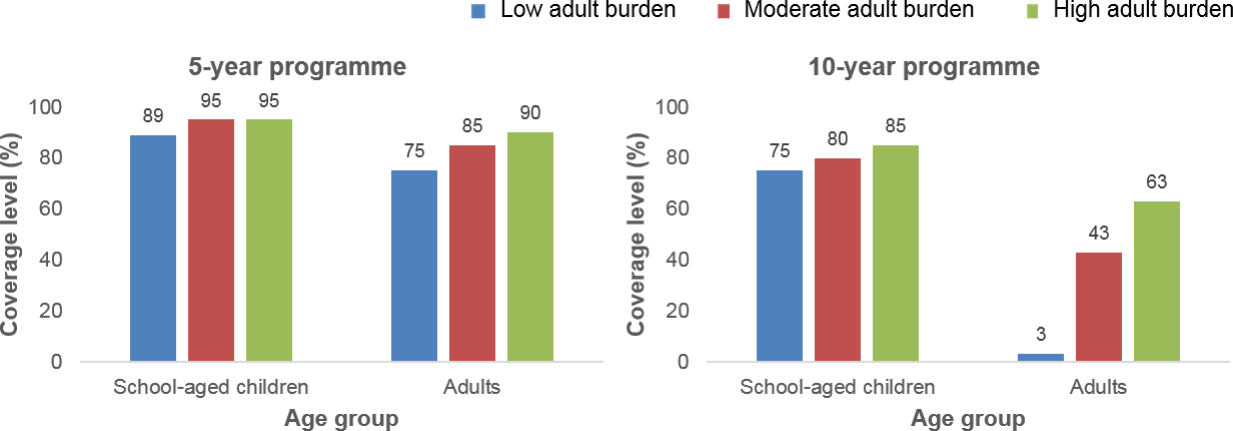
Coverage levels required to reach the World Health Organization (WHO) goal of morbidity control. High prevalence settings (≥50% SAC baseline prevalence) showing coverage levels of school-aged children (SAC; 5–14 years of age) and adults required to reach the WHO goal of morbidity control within 5- and 10-year treatment programmes (see S1A Fig for 15-year treatment programme).

For high prevalence settings, the SAC and adult coverage levels required to reach the WHO goals varied depending on the burden of infection in adults used within the age-intensity profile of infection. Given a low burden of infection in adults, 89% SAC and 75% adults need to be treated to achieve the morbidity goal within 5 years, and 75% SAC only need to be treated to achieve it within 15 years. Given a moderate burden of infection in adults, 95% SAC and 85% adults need to be treated to achieve the morbidity goal within 5 years, and 75% SAC and 25% adults need to be treated to achieve it within 15 years. Given a high burden of infection in adults, 95% of SAC and 90% of adults need to be treated to achieve the morbidity goal within 5 years, and 75% SAC and 60% adults need to be treated to achieve it within 15 years (Figs 2, 5 and S1A).

**Fig 6:**
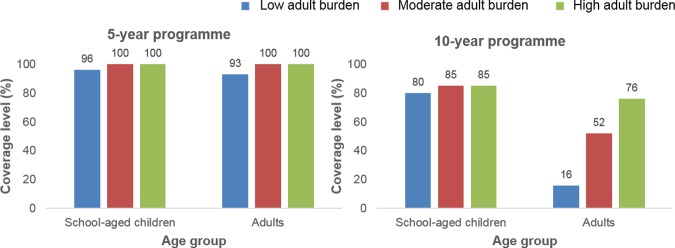
Coverage levels required to reach the World Health Organization (WHO) goal of elimination as a public health problem. High prevalence settings (≥50% SAC baseline prevalence) showing coverage levels of school-aged children (SAC; 5–14 years of age) and adults required to reach the WHO goal of elimination as a public health problem within 5- and 10-year treatment programmes (see S1B Fig for 15-year treatment programme).

The coverage levels increased when the target was the WHO goal of elimination as a public health problem. Given a low burden of infection in adults, 96% SAC and 93% adults need to be treated to achieve the elimination goal within 5 years, and 75% SAC only need to be treated to achieve it within 15 years. Given a moderate burden of infection in adults, all SAC and adults need to be treated to achieve the elimination goal within 5 years, and 75% SAC and 50% adults need to be treated to achieve it within 15 years. Given a high burden of infection in adults, all SAC and adults need to be treated to achieve the elimination goal within 5 years, and 75% SAC and 70% adults need to be treated to achieve it within 15 years (Figs 2, 6 and S1B). Overall, the level of adult coverage required to reach the WHO goals increased as the burden of infection in adults increased (Figs 4–6 and S1). As the number of years of PCT taking place increased, the coverage levels required to achieve the WHO goals decreased as expected.

In summary, the model recommendations for PCT coverage levels vary depending on the age-intensity profile of the region. We have highlighted that the burden of infection in adults as well as SAC will aid in informing the optimal treatment strategy for a region. Hence prevalence and intensity of infection data needs to be collected from adults, in addition to SAC, to inform their burden of infection, both at baseline and during regular surveillance to monitor the treatment programme. More detailed results are shown in S1–S3 Tables.

## Discussion

In this study, we have investigated the impact of the burden of infection in adults on the coverage levels that are required to reach the WHO goals for *S*. *mansoni*. The levels of coverage vary depending on the transmission setting (i.e. R_0_; aligning with previous work [3]). In moderate baseline prevalence settings, the WHO goals of morbidity control and elimination as a public health problem are likely to be achieved following treatment of SAC only, regardless of the burden of infection in adults. However, in high baseline prevalence settings, the untreated reservoir of infection in adults can maintain infection within the whole community making it clear that adult data needs to be collected to inform the optimal treatment coverage levels. Although the prevalence of infection in SAC can be predictive of the burden of infection in adults in some settings, this has been an inconsistent indicator [15–19]. Our results have shown that it is vital that adults are included in M&E programmes, particularly at baseline, as this will inform the optimal PCT strategy for a defined region. We have highlighted additional data requirements in Table 2.

**Table 2:** Data requirements for monitoring and evaluation (M&E) programmes.

**Baseline data**	• Demographic census data on sub-populations (adults, SAC attending school, total SAC) required as denominators in assessing prevalence and treatment coverage [35]. • Mapping data of the region given the spatial heterogeneity of the disease [36]. • Data from low prevalence areas as we move towards elimination since most available data are from higher prevalence areas. • Data sharing among investigators.
**Prevalence and intensity of infection data**	• At baseline and following treatment, cross-sectional and longitudinal infection intensity and prevalence data across all age classes to indicate disease status, produce pre- and post-treatment age profiles of infection and estimate R_0_ [8]. • Following treatment, prevalence of infection may bounce-back but likely at a lower average intensity of infection [37]. • Longitudinal records of egg counts with corresponding data on age and treatment history to record individual intensities of infection over time [24, 38].
**Treatment data**	• Treatment coverage and adherence data at an individual level at each round of treatment to estimate coverage and adherence by age group to determine whether the target coverage level has been achieved [8, 24, 38].
**Water, sanitation and hygiene (WASH) data**	• Another NTD intervention is prevention of infection by improving WASH measures. WASH interventions can be difficult to distinguish from MDA since both are likely to have a similar impact on the prevalence of infection. • Monitoring of facilities to check whether they have been purchased and installed. Individual and/or house-hold behaviour monitored to see if the facilities are being used (can be difficult to measure as behavioural regression may arise).
**Snail data**	• Snail abundance and infection data is required as this is currently lacking.
**Parasite data**	• Parasite life expectancy data in humans as this can range from 3.5 to 10.5 years for schistosomiasis worms [8, 21, 27]. • Data on the nature of density-dependent fecundity [8]. • Autopsy data on parasite loads stratified by sex in infected patients [39].
**Diagnostic techniques and cost data**	• Kato-Katz needed to compare to baseline data, unless CCA can be related to Kato-Katz. • Cost data as costs of the diagnostic tests vary in different settings and costs per test are not constant [14, 40]. • Data on which age groups are infected to assist in determining the most cost-effective treatment strategy [8, 14, 17].

One limitation of this study is that there is currently a lack of data on individual adherence following repeated treatment. At present, no recommendations are made about monitoring adherence to treatment at each round of MDA. Within our model, we have assumed that coverage at each round of treatment is at random, i.e. the individuals receiving treatment in each round are chosen at random. However, if there are persistent non-adherers to treatment (or subgroups such as pregnant women that are often excluded from national treatment programmes), then higher and/or broader age-related coverage levels will likely be required to reach the WHO goals. Hence, collecting longitudinal data on adherence to treatment by individuals within M&E programmes is vital. Another limitation is that using these parameter values, our model is unable to reach a stable equilibrium for low prevalence settings (<10% SAC baseline prevalence). Additionally, we are assuming that the age-intensity profile of infection is formed by the same mechanism following treatment. However, if some of the reduction in prevalence and/or intensity of infection in adults is due to acquired immunity (which may be more relevant when extending this analysis to *S*. *haematobium*), then treatment will also impact on the underlying mechanism that creates the age profile. For example, under the assumptions that acquired immunity is an important determinant of infection in older individuals and treatment of SAC only, this age group may then be less immune as adults, leading to a rise in adult prevalence over time as MDA impact on transmission increases. It is likely that adult treatment would be important and required to prevent this but only knowledge of the dynamics of burdens in adults can reveal this requirement. Consequently, we recommend regular assessment of prevalence and intensity of infection in adults, particularly in cases where the prevalence in SAC does not decline following treatment.

There are logistical difficulties in collecting data from adults. SAC are typically targeted by control programmes as they are relatively easy to reach within school settings and therefore coverage could be high. Some past field studies suggest that treating adults has required more personnel, time, and resources to find and obtain follow-up samples [18]. We note these challenges but recommend collecting adult data, at least at baseline (prior to treatment), to inform PCT and then following each round of treatment if possible. Such challenges could be reduced following effective sensitization within a community. Importantly, the adults should be sampled at random to avoid misleading results. The degree of infection in the adult population and the peak age of infection are key features to capture as the age-profile of infection at baseline will assist in determining which age group to treat for schistosomiasis infection. Such baseline data from a wider range of ages in the population will permit more precise parameterization of the model and hence better predictions of the levels of treatment required to achieve the WHO goals. By not treating infected individuals (including any infected persistent non-adherers to treatment) a reservoir of infection will remain [8, 9] making the WHO goals more difficult to achieve. We have shown that treating SAC only can leave adults heavily infected. Furthermore, it is important that both prevalence and intensity of infection data is collected from adults as well as SAC. The relationship between prevalence and mean intensity of infection is non-linear with the precise relationship depending on the degree of parasite aggregation in the human host population as measured inversely by the negative binomial aggregation parameter (k) [6, 41–43]. Following treatment, prevalence typically bounces-back but at a lower intensity [37].

Diagnostic techniques are becoming increasingly important as prevalence needs to be accurately detected at low levels, particularly as programmes move from morbidity control towards elimination. Hence, it is important to consider which diagnostic techniques will be used in monitoring schistosomiasis infection. The most common diagnostic tools for schistosomiasis are the traditional Kato-Katz diagnostic technique (which current WHO guidelines are based on [2]) and the relatively newer, more sensitive point-of-care circulating cathodic antigen test (CCA) diagnostic test [37, 44, 45]. Although Kato-Katz is seen as the cheaper test, given the increased sensitivity of CCA, this may outweigh costs in long term as the CCA test is faster, less labour intensive and likely to have a higher prevalence threshold [14, 37, 46]. However, the costs vary in different settings and costs per test are not constant [14]. Additionally, there are no current WHO guidelines on mapping with such new diagnostics, nor subsequent treatment cut-offs using more sensitive tools. Economies of scale are important when considering the costs of MDA as this means the cost per treatment decreases as the number of people treated increases [40]. Currently, programmes focus on sampling SAC due to financial and programmatic constraints [14]. Accurate data on which age groups are infected are required to assess for the most cost-effective treatment strategy [14]. Hence, the need for adult inclusion within M&E programmes highlights the need for more cost-effective, rapid and accurate diagnostics [14, 17]. Notably, there is a need to obtain data relating diagnostic techniques (such as Kato Katz and CCA on samples from the same individual at the same time) and more accurate cost data.

We have also highlighted other data that should be collected within M&E programmes, including more accurate, detailed prevalence and intensity of infection data, treatment coverage and adherence data, and water, sanitation and hygiene (WASH) data (Table 2). By designing M&E programmes to collect this information, our models can be better parameterized to determine the projected impact of treatment in an area. Furthermore, with better M&E programmes, we can more accurately assess the progress of treatment programmes and improve recommendations on optimal treatment strategies. Well-documented M&E programmes will allow us to assess PCT strategies and aid in the development of additional improvements.

### Conclusions

For moderate prevalence settings, treating school-aged children only may achieve the WHO goals, regardless of the burden of infection in adults. However, for high prevalence settings, treatment of adults as well as SAC is required within the treatment programme, with coverage levels varying with the burden of infection in adults. Hence, the optimal treatment strategy for a defined region cannot be determined solely by the level of infection in SAC. This highlights that prevalence and intensity of infection data in adults needs to be included within M&E programmes, particularly at baseline but also following treatment when monitoring the impact of treatment. The burden of infection in adults will better inform whether adult treatment is required and the coverage levels which are required to achieve the WHO goals and elimination as we look further to the future.

## Supporting information

S1 Fig Coverage levels required to reach the World Health Organization (WHO) goals. High baseline prevalence settings (≥50% SAC baseline prevalence) showing coverage levels of school-aged children (SAC; 5–14 years of age) and adults required to reach the WHO goals of (a) morbidity control and (b) elimination as a public health problem within a 15-year treatment programme.
(TIF)

S1 Table Low burden setting in adults. Levels of school-aged children (SAC; 5–14 years of age) and adult (≥ 15 years of age) coverage required to meet the WHO goals when following currently recommended WHO treatment frequencies, i.e. for moderate baseline prevalence in SAC, treating once every 2 years; for high baseline prevalence in SAC, treating once a year. Required coverage levels are shown for a 5, 10 and 15-year treatment programme. NA: not achievable unless SAC coverage is increased above 75%.
(DOCX)

S2 Table Moderate burden setting in adults. Levels of school-aged children (SAC; 5–14 years of age) and adult (≥ 15 years of age) coverage required to meet the WHO goals when following currently recommended WHO treatment frequencies, i.e. for moderate baseline prevalence in SAC, treating once every 2 years; for high baseline prevalence in SAC, treating once a year. Required coverage levels are shown for a 5, 10 and 15-year treatment programme. NA: not achievable unless SAC coverage is increased above 75%.
(DOCX)

S3 Table High burden setting in adults. Levels of school-aged children (SAC; 5–14 years of age) and adult (≥ 15 years of age) coverage required to meet the WHO goals when following currently recommended WHO treatment frequencies, i.e. for moderate baseline prevalence in SAC, treating once every 2 years; for high baseline prevalence in SAC, treating once a year. Required coverage levels are shown for a 5, 10 and 15-year treatment programme. NA: not achievable unless SAC coverage is increased above 75%.
(DOCX)
