# Supplementary figures and images for: The design of schistosomiasis monitoring and evaluation programmes: The importance of collecting adult data to inform treatment strategies for Schistosoma mansoni
Source: PLoS Negl Trop Dis. 2018 Oct 8;12(10):e0006717. doi: 10.1371/journal.pntd.0006717 (PMC6175503; doi:10.1371/journal.pntd.0006717)

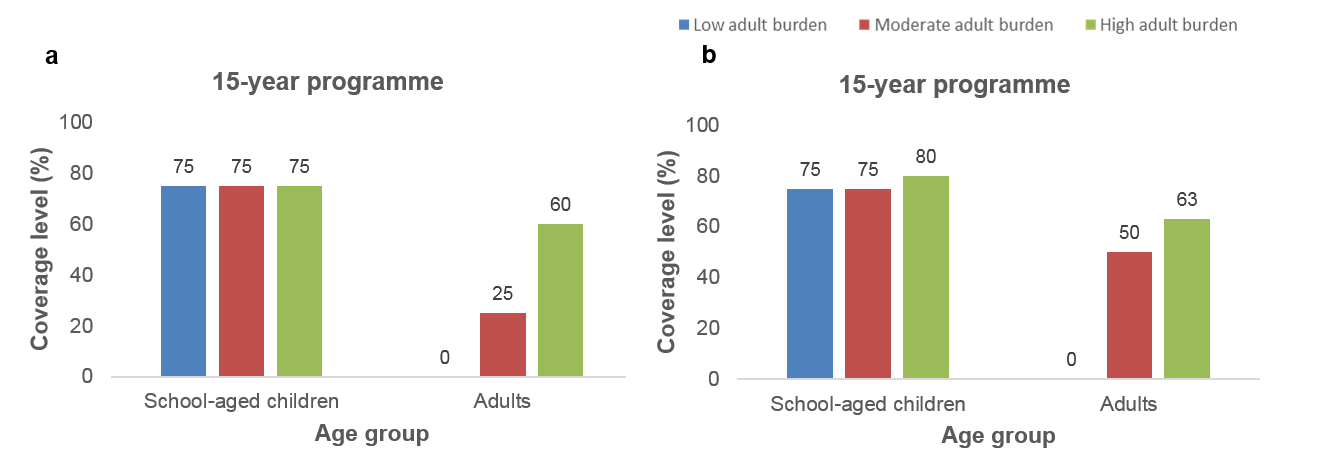

Supplement: S1 Fig — High baseline prevalence settings (≥50% SAC baseline prevalence) showing coverage levels of school-aged children (SAC; 5–14 years of age) and adults required to reach the WHO goals of (a) morbidity control and (b) elimination as a public health problem within a 15-year treatment programme. (TIF) [file pntd.0006717.s001.tif]
