# Supplementary material for: The design of schistosomiasis monitoring and evaluation programmes: The importance of collecting adult data to inform treatment strategies for Schistosoma mansoni
Source: PLoS Negl Trop Dis. 2018 Oct 8;12(10):e0006717. doi: 10.1371/journal.pntd.0006717 (PMC6175503; doi:10.1371/journal.pntd.0006717)
Supplement: S2 Table — Levels of school-aged children (SAC; 5–14 years of age) and adult (≥ 15 years of age) coverage required to meet the WHO goals when following currently recommended WHO treatment frequencies, i.e. for moderate baseline prevalence in SAC, treating once every 2 years; for high baseline prevalence in SAC, treating once a year. Required coverage levels are shown for a 5, 10 and 15-year treatment programme. NA: not achievable unless SAC coverage is increased above 75%. (DOCX) [file pntd.0006717.s003.docx]

**S2 Table. Moderate burden setting in adults.**

| **Baseline prevalence in SAC** | **Goal** | |
| --- | --- | --- |
|  | Morbidity control  (≤5% heavy-intensity infections in SAC) | Elimination as a public health problem (≤1% heavy-intensity infections in SAC) |
| Moderate (10-50%)  Baseline prevalence in SAC: 11.3 – 49.7%  Baseline prevalence in adults: 9.2 – 42.1%  R_0_ values: 1.23 - 1.209  k values: 0.04 - 0.24 | 5/10/15-year programme  SAC: 75%  Adults: 0% | 5/10/15-year programme  SAC: 75%  Adults: 0% |
| High (≥50%)  Baseline prevalence in SAC: 50.3 – 76.2%  Baseline prevalence in adults: 42.8 – 72.1%  R_0_ values: 1.211 - 3.5  k value: 0.24 | 5-year programme  SAC: 75%  Adults: 0% - NA  (SAC 95% + adults 85%)  10-year programme  SAC: 75%  Adults: 0% - 86%  (or SAC 80% + adults 43%)  15-year programme  SAC: 75%  Adults: 0% - 25%  (or SAC 90% + adults 0%) | 5-year programme  SAC: 75%  Adults: 0% - NA  (SAC 100% + adults 100%)  10-year programme  SAC: 75%  Adults: 0% - NA (SAC 85% + adults 52% or SAC 100% + adults 0%)  15-year programme  SAC: 75%  Adults: 0% - 50% |

Levels of school-aged children (SAC; 5-14 years of age) and adult (≥ 15 years of age) coverage required to meet the WHO goals when following currently recommended WHO treatment frequencies, i.e. for moderate baseline prevalence in SAC, treating once every 2 years; for high baseline prevalence in SAC, treating once a year. Required coverage levels are shown for a 5, 10 and 15-year treatment programme. NA: not achievable unless SAC coverage is increased above 75%.
